# Unable to Describe My Feelings and Emotions Without an Addiction: The Interdependency Between Alexithymia and Addictions

**DOI:** 10.3389/fpsyt.2020.543346

**Published:** 2020-10-23

**Authors:** Laura Orsolini

**Affiliations:** ^1^ Unit of Clinical Psychiatry, Department of Neurosciences/DIMSC, School of Medicine, Polytechnic University of Marche, Ancona, Italy; ^2^ Psychopharmacology, Drug Misuse and Novel Psychoactive Substances Research Unit, School of Life and Medical Sciences, University of Hertfordshire, Hatfield, United Kingdom

**Keywords:** addiction, addiction disorders, alexithymia, emotional regulation difficulties, emotional regulation

## Introduction

Alexithymia (from the greek αλϵξιθυμία: α-(a) = absence, λέξις (lexis) = word, θυμός (thymos) = emotion) is a specific form of emotional dysregulation, characterized by difficulty in identifying, describing, and communicating emotions as well as in discriminating one’s own emotional experiences from the underlying ‘physiological activation’ (1). Alexithymia also comprises a cognitive style oriented towards an external reality, poor imaginative processes, and low levels of introspection and pragmatic thinking (1). However, competing hyperarousal and hypoarousal models of alexithymia have been proposed (2). Individuals with alexithymia may display poorly regulated emotions due to a state of hyperarousal related to the deficits in identifying and/or describing one’s own emotions, or in adequately reacting to or modulating them (3). According to the hyperarousal model, alexithymic subjects also display a reduced activation in brain regions related to mentalizing capabilities (i.e., the temporo-parietal junction and dorsomedial pre-frontal cortex) (3). Some authors argue that a hypoarousal model (e.g. characterized by a low autonomic arousal) may explain the increased risk of substance abuse amongst alexithymic subjects (2, 4). In particular, according to the hypoarousal model, alexithymic subjects are more prone to use substances in an attempt to optimize these inherently low arousal levels (2, 4). Moreover, alexithymia has been demonstrated to be closely related to the concept of emotional intelligence (5, 6) and an inverse relationship exists between alexithymia levels and the ability to be resilient to negative life events (7). Furthermore, a potential role of dysfunctional primary parental bonding experiences in childhood and subsequent insecure adult attachment and alexithymia development has been proposed (8). The construct of alexithymia has been largely defined, as shown in **Table 1** (7, 9).

**Table 1:** The construct of alexithymia: main features.

• Difficulty in identifying feelings and in distinguishing them from bodily sensations that accompany emotional activation
• Difficulty in describing own feelings to others
• Limited imaginative processes, highlighted by the poverty of imagination and fantasy thought
• Cognitive style related to the stimulus and externally oriented
• Strict adherence to the rules, as if the identity is defined only from the outside and external reality
• Lack of introspective abilities and lacking mentalizing processes
• Inability to recognize and express emotions even at a non-verbal level
• Lack of emotional activation even in the face of emotionally meaningful events
• Lack of relational skills, poor tolerance to stress, lack of humor and excessive self-control

The identification and evaluation of an impaired emotional regulation have been widely linked to the etiology and treatment of substance use disorders (SUDs), alcohol use disorders (AUDs), and behavioural addictions (10). The incapability to modulate emotions, due to a cognitive elaboration, may explain the higher levels of impulsivity and compulsive behaviors (i.e. binge eating, drug and/or alcohol addiction, sex addiction, internet addiction, and gambling) which have been observed amongst alexithymic subjects. In addition, individuals with alexithymia appear to have difficulties in addressing stressful situations and are more prone to use ‘addictive’ modalities as a coping strategy for managing potentially distressing situations (11). In fact, alexithymia has been widely associated with increased cannabis use in adolescence, increased caffeine consumption, and increased development of mobile phone addiction and/or pathological gambling (10–17).

## Substance and Alcohol Use Disorder and Alexithymia

Alexithymia may be a risk factor for SUDs, particularly in the context of AUDs (13, 14). The relationship between alexithymia and addiction is supported by significant findings describing a correlation between alexithymic traits and craving, severity of SUD and/or AUD, and related complications (18, 19). Emotional dysregulation has been widely associated with alexithymia in SUD and/or AUD (3). Individuals with illicit drug use frequently (45% to 50%) exhibit alexithymia (20, 21). Research suggests a correlation between alexithymia and craving for drugs and/or alcohol (22–24). Around 45% to 67% of subjects with AUD reported high alexithymia scores (23). Moreover, alexithymia has been associated with a family history of alcoholism (24). In social drinkers, alexithymia may predict alcohol consumption (25). Several mechanisms have been proposed to explain the neurobiological basis of alexithymia in addiction, from an aberrant hypothalamic-pituitary-adrenal axis functioning associated with chronic stress, to the lack of interoceptive and emotional awareness which may prevent effective emotion regulation and lead an individual to use a substance addiction as a coping strategy to alleviate distress (26–29). Furthermore, some findings reported an anxious attachment style, alcohol expectancy, and drinking motives as mediating factors in AUDs (18, 19). Moreover, inadequate parental bonding during infancy and childhood may result in deficits of self-regulation, emotion recognition, and an insecure interpersonal adult attachment, which may encourage reliance on substances and/or alcohol as emotional disinhibiting and/or alleviating tools in response to negative effects in alexithymic subjects (8).

## Pathological Gambling and Alexithymia

Pathological gambling and gambling disorder are two terms often used interchangeably, characterized by financial, psychological, employment, and interpersonal difficulties related to excessive wagering (30). Gambling disorders may lead to legal issues and suicidal ideation and behaviours (31). Several risk factors have been hypothesized, including socio-cultural background, personality factors (i.e. impulsivity, sensation seeking, antisocial behaviours, etc.), cognitive distortions, and an impairment of emotional processes (32, 33). Amongst the different personality traits thought to be implicated in the development and maintenance of gambling disorders, the construct of alexithymia and an affect dysregulation, i.e. the inability to tolerate negative affect, have been further investigated (34). Alexithymia is commonly represented in subjects with gambling-related problems by causing a worsening in symptom severity and increasing the risk of pathological gambling (34). The difficulty in identifying one’s own emotional state may impair the decision-making process, which is particularly relevant in gambling disorders. Thus, the subject is unable to use the information contained in an aversive (negative) emotional state related to a negative external feedback due to impaired emotional regulation (35). Furthermore, alexithymia displays a clinically significant interaction with maladaptive personality (e.g. impulsivity, aggressiveness, and sensation-seeking personalities), cognitive factors (e.g. gambling-related cognitive distortions, motivation, strategic and non-strategic games) as well as psychopathological symptomatology, including depression and anxiety (34).

## Internet Addiction and Alexithymia

Internet Addiction (IA) is characterized by staying on the Internet for longer than planned and experiencing withdrawal symptoms such as nervousness, low mood, or restlessness when the subject is deprived of the Internet (36, 37). The symptomatology is often accompanied by diminished sleep quality and hygiene, relational maladjustment, and progressive worsening of personal, family, social and occupational/school functioning due to excessive Internet use and consequent cognitive and psychological impairment(s) (36, 37). IA has been associated with several psycho-social determinants/risk factors, including impaired emotional regulation and higher levels of alexithymia, particularly amongst youngsters/adolescents (37–40). Overall, adolescents with IA appear to have more difficulties in identifying and describing emotions, understanding emotional reactions, and controlling their impulsive behaviours in response to negative emotional experiences (mainly due to an inadequate prefrontal cognitive control). Moreover, adolescents with an IA appear to be less able to use effective strategies in emotional regulation compared to adolescents without an IA (41–43). Similarly, subjects with higher levels of alexithymia are more likely to develop an IA compared to non-alexithymic subjects (38, 39, 44).

## Mobile Phone Addiction and Alexithymia

Mobile phone addiction is a behaviour characterized by an excessive indulgence to activities related to mobile phones, accompanied by a constant dependence on mobile phones, which results in a general loss of self-control and compromised psychological and social functioning (44–46). Mobile phone addicts might possess emotional recognition deficits, which may lead to more interpersonal issues as they usually attempt to cover/mask their own emotional states by escaping from reality by means of mobile phones (9). Alexithymic subjects may use online gaming as a coping strategy for their distressing psychosocial issues in real life (9, 47).

## Internet Gaming Disorder and Alexithymia

A failure of emotional regulation and control associated with high levels of alexithymia have been demonstrated by several studies which investigated the features underlying the development of a pathological gaming (12, 48, 49). Alexithymia might explain the engagement in and the maintenance of video game use amongst those individuals with poorly regulated emotions. In fact, these individuals often engage in maladaptive behaviours, including internet gaming disorders, to escape from or downregulate their own emotions (35, 49).

## Discussion

Currently, alexithymia is mainly considered as a personality construct, characterized by a marked dysfunction in emotional awareness, social attachment, and interpersonal relating (50). However, some authors suggest that alexithymia might represent a state-dependent mechanism (as in, something which is temporary and situation-dependent, i.e. secondary alexithymia) which may develop as a reaction to psychological distress (50, 51). Taylor et al. (51) demonstrated that alexithymic subjects generally attempt to regulate their emotions through compulsive and/or impulsive behaviours (e.g., SUDs, AUDs, and behavioural addictions) (3). Other studies suggest that alexithymia might be considered a state-dependent mechanism in patients addicted to substances and/or behaviours (13), hence, secondary to an immature/maladaptive defense style used by these patients, in an attempt to relieve negative emotions, such as anxiety or depression (50). Further studies support the hypothesis that alexithymia might represent a stable personality trait, greatly influenced and caused by several factors, including life experiences, previous relationships, early traumatic experiences (e.g. childhood maltreatment and/or abuse), and the type of attachment system (e.g. insecure attachment), which may, in turn, cause increased vulnerability to addictive disorders (9, 18, 52).

Failures in emotional regulation, observed in alexithymic subjects, makes it difficult for them to discern, assess, and express their feelings and to understand others’ feelings/emotions in face-to-face communication (11). These core features of alexithymia may cause an inadequate emotional response and a perceived lack of empathy, which, in turn, may cause a failure to establish and maintain ‘face-to-face’ relationships in the real world (11). Therefore, the feeling of not being accepted and the lack of a sense of belonging might lead alexithymic subjects to use alternative modalities to communicate and interact with others (e.g. online platforms, Internet, and mobile phones) which may result in the early development of a technological addiction (46). Overall, one could argue that alexithymia might be both a stable personality trait that may constitute a vulnerability/risk factor for the development and/or maintenance of an addiction disorder, and a defensive mechanism (i.e., a variable state) secondary to a psychological distress which may arise or worsen an SUD/AUD or a behavioural addiction already present (3, 53).

Moreover, the early identification and assessment of the presence of an alexithymic dimension (trait or state) may help clinicians to adequately assign patients to specific treatments associated with better outcomes, such as computerized cognitive behavioural therapy (3, 54). In this regard, clinicians working in addiction services should be aware of the current validated instruments and scales designed to assess alexithymic traits and/or states in medical and psychiatric research (55–57), especially amongst addictive subjects (58–62). Presently, the Toronto Alexithymia Scale-20 items (TAS-20) and the Bermond-Vorst Alexithymia Questionnaire (BVAQ) are the most known, reliable, and validated alexithymia self-report tools, also commonly used in the addiction field (58, 59, 61, 63, 64). However, several studies have specifically investigated the psychometric properties of further alexithymic self-reports or observer-rated tools in clinical samples with addiction disorders (60, 62). Amongst these rating scales, researchers suggest also using the Observer Alexithymia Scale (OAS, 33-item, observer-rated scale) (62, 65).

Alexithymia may interfere with treatment and prognosis outcomes in the addiction context (62). In fact, those individuals for whom it is difficult to recognize and describe emotional states may not be able to adequately regulate their emotional states or recognize their relationship to the initiation and/or the maintenance of a substance/alcohol and/or a behavioral addiction, especially in a state of heightened distress (66–69). Alexithymia may also predict treatment engagement and adherence, in terms of session attendance and working alliance (58) and may cause higher rates of relapse and premature treatment discontinuation (70). However, the evidence so far generated is limited and non-univocal. In fact, de Haan et al. (71) did not report any differences in mean time in treatment or dropout rates amongst SUD subjects with high-scoring vs low-scoring alexithymia, both measures being continuous and categorical variables. Similarly, alexithymia was not strongly associated with treatment adherence or retention in an 8-week randomized clinical trial (54, 72).

Overall, clinicians should be aware of the possible interdependent relationship between the presence of alexithymic dimensions (trait or state) and the development, maintenance, and/or worsening of an addictive disorder, in order to better assemble and perform personalized treatment protocols, which also take into account the dimension of alexithymia. Furthermore, although the “chicken or the egg causality dilemma” remains unresolved (i.e. if alexithymic dimension is a trait or state), clinicians should always investigate the presence of alexithymic traits of personality or alexithymic state which could cause different outcomes and prognoses in the context of an addiction. Moreover, in the context of the recent growth and dissemination of digital platforms and the subsequent development of intriguing, potentially addictive and appealing online modalities amongst youngsters, clinicians should consider the possibility of a relationship between the presence of an alexithymic trait/state and the subsequent occurrence of IA, mobile phone dependence, and/or online gaming disorder amongst adolescents. Finally, one could argue that an alexithymic dimension is likely becoming a ‘quasi-pandemic’/’quasi-physiologic’ trait of modern adolescence, in the light of the increasing incidence of several co-occurring changes which may affect individual, social, and family contexts. Therefore, clinicians should consider the preventive strategies (if any) and therapeutic approaches needed in the concurrent presence of alexithymic traits/states in the context of an addiction. Further research should address and further develop this issue in the context of addiction.

## Author Contributions

The author confirms being the sole contributor of this work and has approved it for publication.

## Conflict of Interest

The author declares that the research was conducted in the absence of any commercial or financial relationships that could be construed as a potential conflict of interest.
